# Assessment of Changes in US Veterans Health Administration Care Delivery Methods During the COVID-19 Pandemic

**DOI:** 10.1001/jamanetworkopen.2021.29139

**Published:** 2021-10-14

**Authors:** Liam Rose, Linda Diem Tran, Steven M. Asch, Anita Vashi

**Affiliations:** 1 Health Economics Resource Center, Veterans Affairs Palo Alto Health Care System, Menlo Park, California; 2 Stanford Surgery Policy Improvement and Education Center, Stanford Medicine, Stanford University, Stanford, California; 3 Center for Innovation to Implementation, Veterans Affairs Palo Alto Health Care System, Menlo Park, California; 4 Division of Primary Care and Population Health, Stanford Medicine, Stanford University, Stanford, California; 5 Department of Emergency Medicine, University of California, San Francisco

## Abstract

This cross-sectional study assesses changes in health care delivery methods, including all forms of care either purchased or provided by the Veterans Health Administration (VHA), for VHA enrollees in response to the COVID-19 pandemic.

## Introduction

The Veterans Health Administration (VHA) manages an integrated health care system that has expenditures of nearly $100 billion per year and serves more than 9 million enrollees.^1^ Like other health care systems, the VHA has faced unprecedented challenges in responding to the COVID-19 pandemic. Although its large size, diverse operating environments, and geographically dispersed patient population make it difficult for the VHA to pivot nimbly and ensure access to care, this health system was able to leverage its existing infrastructure and prior planning to rapidly scale virtual care services (ie, telephone and video) for enrollees in 2020.^2,3^ In this study, we took a broad look at how VHA care patterns, including all forms of care either purchased (known as community care) or provided by the VHA, have shifted in association with the COVID-19 pandemic.

## Methods

For this cross-sectional study, we extracted records from the US Department of Veterans Affairs Corporate Data Warehouse on all health care encounters purchased or provided by the VHA from January 1, 2019 to March 28, 2021. Encounters were then classified into mutually exclusive categories by location (VHA and community care) and type of care delivered (inpatient, emergency department, urgent care, and outpatient). Patient age, sex, and race and ethnicity demographics were collected from the records to assess whether they reflected the general population of VHA enrollees. Further details on data aggregation are provided in the eMethods in the Supplement.

Encounter data were aggregated by epidemiologic weeks. To estimate the total number of missing encounters in 2020, we performed local polynomial regression of total weekly encounters on prepandemic 2019 numeric weeks and applied the smoothed values (ie, expected total encounters per week if prior levels had persisted) to 2020 numeric weeks. We subtracted the smoothed values from actual 2020 totals and summed the differences across all weeks.

This study followed the Strengthening the Reporting of Observational Studies in Epidemiology (STROBE) reporting guideline. The Stanford University Institutional Review Board approved the study. This was an observational study without any direct patient contact and was considered to be of minimal risk; therefore, a waiver of informed consent was obtained.

## Results

The VHA provided or paid for 179.5 million encounters for 6 737 274 unique patients between January 2019 and March 2021. The study demographics reflected the general VHA enrollee population: the median age was 66 (SD, 17) years; 5 808 690 (86.2%) were men and 928 584 (13.8%) were women; and 50 564 (0.007%) were American Indian, 131 155 (1.9%) were Asian or Pacific Islander, 1 094 073 (16.2%) were Black, 4 530 293 (69.2%) were White, and 31 145 (4.6%) were multiple races or were of unknown race or ethnicity.^1^ The Figure provides the number of encounters over time by location and type of care delivered. As expected, overall health care use decreased substantially in March and April 2020 decreased substantially (1.97 million encounters in the last week of February 2020 vs 1 million in the first week of April 2020), and virtual care expanded swiftly (90 400 encounters in the last week of February 2020 vs 404 000 encounters in the first week of April 2020). Strikingly, the number of total encounters has not yet recovered to prepandemic levels (1.97 million encounters per week in February 2020 vs 1.7 million per week in February 2021). The estimated total number of missing encounters relative to the previous year was 16.5 million.

**Figure.**
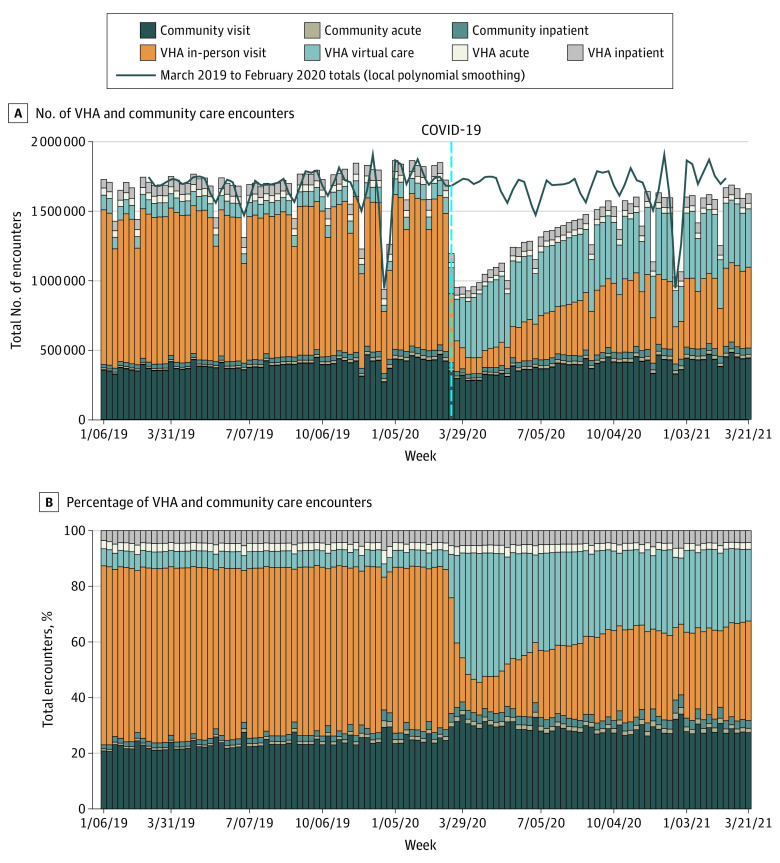
All VHA and Community Care Encounters, January 2019 to March 2021 Numbers (A) and percentages (B) of all health care encounters purchased (community care) or provided by the US Veterans Health Administration (VHA). Acute care refers to inpatient, emergency department, and urgent care encounters. Virtual care refers to both telephone and video encounters.

The Table describes the share of each care category, along with the percent change, for select months. The number of VHA virtual care encounters increased from 454 399 (6.0%) in April 2019 to 1 894 674 (44.6%) in April 2020, then decreased to 1 861 922 (28.8%) in December 2020. The number of nonacute community care encounters was 1 676 398 (22.2%) in April 2019 compared with 1 292 029 (30.4%) in April 2020, which remained steady as of December 2020 (1 868 029 [28.9%]). As of December 2020, VHA in-person care constituted just 29.7% of VHA purchased or provided care, with 1 918 513 encounters.

**Table.** Changes in Community Care and VHA Encounters During the COVID-19 Pandemic and Relative to 2019

	April 2019 to April 2020			December 2019 to December 2020		
	No. (%) of encounters^a^		Change from 2019 to 2020, %^b^	No. (%) of encounters		Change from 2019 to 2020, %
	April 2019	April 2020		December 2019	December 2020	
No. of total encounters	7 563 934	4 252 835	−43.8	6 917 780	6 461 926	−6.6
Community care						
Inpatient	129 442 (1.7)	129 842 (3.1)	0.3	173 600 (2.5)	220 764 (3.4)	27.2
Acute	57 424 (0.8)	66 977 (1.6)	16.6	98 120 (1.4)	112 492 (1.7)	14.6
Encounters	1 676 398 (22.2)	1 292 029 (30.4)	−22.9	1 747 334 (25.3)	1 868 029 (28.9)	6.9
VHA care						
Inpatient	327 675 (4.3)	221 389 (5.2)	−32.4	329 153 (4.8)	309 423 (4.8)	−6.0
Acute	214 414 (2.8)	115 097 (2.7)	−46.3	209 317 (3.0)	170 783 (2.6)	−18.4
In person	4 704 182 (62.2)	532 827 (12.5)	−88.7	3 957 823 (57.2)	1 918 513 (29.7)	−51.5
Virtual^c^	454 399 (6.0)	1 894 674 (44.6)	317.0	402 433 (5.8)	1 861 922 (28.8)	362.7

Abbreviation: VHA, Veterans Health Administration.

^a^ Proportion of all encounters purchased or provided by the VHA in that category in 2019 and 2020.

^b^ Percent change in care delivery method (*z* score).

^c^ Virtual care refers to both telephone and video encounters.

## Discussion

In this cross-sectional study of health care use patterns of VHA enrollees, we observed substantial and persistent changes in the number of virtual and community care encounters over the year since the start of the COVID-19 pandemic in 2020. Limitations of this study include a likely lag in adjudication of more recent community care claims, which indicates that current estimates of community care encounters may be underestimated.

Like other systems, the VHA experienced large reductions in care early in the pandemic and was well positioned to quickly transition to providing virtual care.^2,3^ However, the number of encounters for VHA in-person care declined substantially more than that for community care encounters and has not yet recovered. Our results indicate that the VHA has likely adopted a more conservative reopening strategy compared with community care providers. These providers tend to have different financial incentives to resume in-person care, and many returned to close to prepandemic inpatient and outpatient levels by September 2020.^4,5^ In the wake of concerns about health care access, the VHA has steadily increased spending on community care, and our results indicate that existing trends pushing the VHA toward being a mixed payer and provider may have accelerated.

## Notes

Supplement. **eMethods.** Data Aggregation
