# Supplementary material for: Assessment of Changes in US Veterans Health Administration Care Delivery Methods During the COVID-19 Pandemic
Source: JAMA Netw Open. 2021 Oct 14;4(10):e2129139. doi: 10.1001/jamanetworkopen.2021.29139 (PMC8517744; doi:10.1001/jamanetworkopen.2021.29139)
Supplement: Supplement. — eMethods. Data Aggregation [file jamanetwopen-e2129139-s001.pdf]

Supplementary Online Content

Rose L, Tran LD, Asch SM, Vashi A. Assessment of changes in US Veterans Health Administration care delivery methods during the COVID-19 pandemic. *JAMA Netw Open*. 2021;4(10):e2129139. doi: 10.1001/jamanetworkopen.2021.29139

eMethods. Data Aggregation

This supplementary material has been provided by the authors to give readers additional information about their work.

© 2021 Rose et al. *JAMA Network Open*.

eMethods. Data Aggregation

Data Sources
VA data were extracted on August 2, 2021 from VA records housed in the VA Corporate Data Warehouse (CDW) to include all visits from January 1, 2019 to the last full week of March 2021 (March 28, 2021). We included records that come from VA health records as well as Community Care claims data from the Program Integrity Tool (PIT) following best practices as provided by the VA Office of Community Care.

Defining Visit Types
VA inpatient visits: All VA inpatient encounters, excluding long-term care stays, were extracted from CDW Inpatient tables using bed sections. For inpatient stays lasting multiple days, a visit was counted for each day of the stay.

VA ED and UC visits: VA ED and UC encounters were defined by stop codes used at eligible facilities and were aggregated by unique patient and date of service. VA acute visits comprised of VA ED and VA UC visits. Multiple ED and UC visits on the same day were only counted once.

VA outpatient visits: Outpatient encounters were defined as completed appointments in “count” clinics and excluded ED and UC visits. Multiple appointments in the same location/clinic on the same day were categorized as one visit. In-person and virtual outpatient visits were defined by primary and secondary stop codes.
Community Care inpatient visits: Inpatient stays were identified by revenue codes. For inpatient stays lasting multiple days, a visit was counted for each day of the stay.

Community Care ED and UC visits: We used procedure codes and revenue codes available from Program Integrity Tool (PIT) claims to identify Community Care ED visits and aggregated visits by unique patient and preauthorization number, and then by patient and date of service, as recommended by the VA Office of Community Care. Non-VA UC visits were defined by place of service and/or preauthorization number.

*Community Care outpatient visits: O*utpatient encounters included unique non-ED, non-UC, and non-inpatient claims. We categorized visits by unique patient, preauthorization number, and date of service. While outpatient visits were not further categorized as in-person or virtual, both types were included in totals, as virtual Community Care encounters were less than 0.1% of all non-acute Community Care encounters.

© 2021 Rose et al. *JAMA Network Open*.
